# Insights into the global freshwater virome

**DOI:** 10.3389/fmicb.2022.953500

**Published:** 2022-09-28

**Authors:** Ali H. A. Elbehery, Li Deng

**Affiliations:** ^1^Department of Microbiology and Immunology, Faculty of Pharmacy, University of Sadat City, Sadat City, Egypt; ^2^Helmholtz Centre Munich – German Research Centre for Environmental Health, Institute of Virology, Neuherberg, Germany; ^3^Chair of Microbial Disease Prevention, School of Life Sciences, Technical University of Munich, Freising, Germany

**Keywords:** freshwater, virome, metagenome, bacteriophages, auxiliary metabolic genes

## Abstract

Viruses are by far the most abundant life forms on this planet. Yet, the full viral diversity remains mostly unknown, especially in environments like freshwater. Therefore, we aimed to study freshwater viruses in a global context. To this end, we downloaded 380 publicly available viral metagenomes (>1 TB). More than 60% of these metagenomes were discarded based on their levels of cellular contamination assessed by ribosomal DNA content. For the remaining metagenomes, assembled contigs were decontaminated using two consecutive steps, eventually yielding 273,365 viral contigs longer than 1,000 bp. Long enough contigs (≥ 10 kb) were clustered to identify novel genomes/genome fragments. We could recover 549 complete circular and high-quality draft genomes, out of which 10 were recognized as being novel. Functional annotation of these genomes showed that most of the annotated coding sequences are DNA metabolic genes or phage structural genes. On the other hand, taxonomic analysis of viral contigs showed that most of the assigned contigs belonged to the order *Caudovirales*, particularly the families of *Siphoviridae*, *Myoviridae,* and *Podoviridae*. The recovered viral contigs contained several auxiliary metabolic genes belonging to several metabolic pathways, especially carbohydrate and amino acid metabolism in addition to photosynthesis as well as hydrocarbon degradation and antibiotic resistance. Overall, we present here a set of prudently chosen viral contigs, which should not only help better understanding of freshwater viruses but also be a valuable resource for future virome studies.

## Introduction

Viruses are the most numerous biological entities on Earth, with a global estimate of 4.80 × 10^31^ virus like particles (VLPs; Güemes et al., 2016). Freshwater is estimated to contain 1.76 × 10^27^ VLPs, which represents 0.0037% of VLPs on the globe. This number of VLPs in freshwater outnumbers the total number of prokaryotes in freshwater by 14 times (Güemes et al., 2016). Most of these viruses are viruses which infect prokaryotes (Edwards and Rohwer, 2005). Based on the mass of an average phage with a 50 kb genome and an icosahedral capsid, which is 0.0823 femtograms (Güemes et al., 2016), the total mass of viruses in freshwater could be estimated to 144,848 tons, which is more than the weight of 1,800 blue whales; the weight of an average mature female blue whale is 79 tons (Krogh, 1934). In comparison, marine ecosystems are estimated to contain 1.29 × 10^30^ VLPs (Güemes et al., 2016), which is almost three orders of magnitude higher than VLPs in freshwater. Yet, viral communities are distinct between these two ecosystems (Logares et al., 2009; Roux et al., 2012), denoting biome-specific diversity.

Phages play important ecological roles. For example, phages, through lysis of bacteria, contribute to carbon cycling, where dissolved organic matter released from lysed bacterial cells become available to other bacteria in a process known as viral shunt (Wilhelm and Suttle, 1999). In addition, the ability of phages to kill bacteria allow them to regulate and control the size and diversity of microbial communities. One of the most popular models by which viruses are believed to control microbial populations, is “killing the winner,” where viruses selectively kill abundant bacterial taxa, allowing rare taxa to grow and bringing back balance between bacteria taxa in the ecosystem (Thingstad and Lignell, 1997). Such model was previously reported in studies concerned with freshwater viruses, where bacterial diversity increased with increases in viral abundance (Auguet et al., 2009; Meunier and Jacquet, 2015). Moreover, viruses contribute to the functional diversity of microbial communities through gene transfer, which occurs through transduction, where chromosomal or plasmid DNA can be transferred among bacteria by means of viruses (Morrison et al., 1978; Ripp et al., 1994; Replicon et al., 1995; Kenzaka et al., 2010). Interestingly, many phages own host genes that are expressed during infection to overcome host metabolic bottlenecks and enhance viral production. These genes are known as auxiliary metabolic genes (AMGs; Thompson et al., 2011). AMGs span a wide range of metabolic processes, including photosynthesis (Ruiz-Perez et al., 2019), carbon metabolism (Hurwitz et al., 2013), nucleic acid synthesis (Chevallereau et al., 2016), nitrogen metabolism (Gazitúa et al., 2020), sulfur metabolism (Mara et al., 2020) and other metabolic pathways (Warwick et al., 2019). Although most virome studies have been concerned with marine environments, there is an increasing effort to study viruses in freshwater (Jacquet et al., 2010), assessing viral communities in diverse freshwater biomes. They addressed different aspects, such as the prevalence of *Podoviridae* and *Siphoviridae* in the largest lake in Ireland (Skvortsov et al., 2016), the characteristics and prevalence of particular phage, infecting *Fonsibacter*, a bacterioplankton abundant in freshwater ecosystems (Chen et al., 2019), new roles of freshwater viruses in carbon fixation and methylotrophy (Coutinho et al., 2020). Yet, freshwater viruses diversity and function are completely far from being fully known.

In this study, we aimed to collect publicly available freshwater viral metagenomes from all across the globe to get a better understanding of viruses in freshwater. We assembled sequence reads and were able to decontaminate them, relying on both sequence similarity and a machine learning-based tool, thus eventually obtaining viral contigs. We studied the abundance, novelty, function and taxonomy of these contigs in an effort to improve our knowledge of viruses in this virtually untapped environment.

## Materials and methods

### Downloading publicly available metagenomes

Freshwater viral metagenomes were downloaded from the National Center for Bioinformatics Information (NCBI) Sequence Read Archive (SRA)^1^ using SRAdb R package (Zhu et al., 2013) on March 1, 2018, while search_terms = freshwater AND (virus OR viral OR virome OR phage OR phageome) AND (metagenome OR “metagenomic”). These search criteria resulted in 1474 records, but visual inspection of metadata for non-specific results reduced the number of viral metagenomes to 380 (Supplementary Table S1).

### Quality control of sequence reads

Quality control of viral metagenomes was performed by first removing adapters using Cutadapt v.1.16 (Martin, 2011). Cutadapt was also used for N-end trimming (--trim-n) and quality trimming from both ends to a Phred score of at least 15 (--q 15,15). Moreover, all reads with more than two ambiguous nucleotides (N) and/or shorter than 50 nucleotides were removed (--max-n 2, −m 50). Prinseq v.0.20.4 (Schmieder and Edwards, 2011b) was used for the removal of low complexity sequences (--lc_threshold 50, --lc_method entropy), dereplication (--derep 12345) and filtering sequences with noniupac characters and/or with an average Phred score lower than 20 (--noniupac, --min_qual_mean 20). Metagenomes were then filtered from sequences matching PhiX 174 bacteriophage [a known control in Illumina sequencing and a potential contaminant (Mukherjee et al., 2015)], if any, using Deconseq v.0.4.3 (Schmieder and Edwards, 2011a) using default parameters.

### Sequence reads assembly

Illumina reads that passed quality control were assembled, each sample separately, using megahit v1.1.1 (Liu et al., 2015) with the parameters (-t 20 -m 0.8 --preset meta-sensitive), whereas 454 reads were assembled using Newbler v.2.9 (Roche) using default parameters, since Newbler has better performance than other assemblers for Roche 454 reads (Kumar and Blaxter, 2010). Produced contigs were renamed according to the following pattern: freshwater_SRRAccession.contig000XXXXXX, where SRRAccession is the SRA Run accession number of the particular sample used for assembly and XXXXXX is a serial number. Metagenomes that produced no contigs longer than or equal to 1,000 bp were removed from further analysis.

### Assessing and removing cellular contamination

Contamination of viral metagenomes (viromes) with cellular sequences is unavoidable (Roux et al., 2013). A diagram explaining the assessment and removal of cellular contamination is shown in Supplementary Figure S1. We assessed the level of cellular contamination by detection of ribosomal DNA reads using meta_rna (Huang et al., 2009), which reports reads matching 5S, 16S and 23S ribosomal genes. We also sought to remove cellular-like sequences without false positive removal of true viral sequences. Hence, we downloaded Refseq Archaea, Bacteria, Fungi and Protozoa on April 19, 2018 (release March 12, 2018).^2^ We also downloaded Refseq and non-Refseq prokaryotic viruses from NCBI as previously described (Grazziotin et al., 2017; Elbehery et al., 2018). Moreover, we predicted prophages from RefSeq bacterial and archaeal genomes (complete genomes only; 9,355 bacterial and 283 archaeal genomes) using PhiSpy v.2.3 with default parameters (Akhter et al., 2012). Predicted bacterial and archaeal prophages were shredded using the Shred tool from the BBTools suite [Joint Genome Institute (JGI)]^3^ with a fragment length of 80, an overlap of 40 and a minimum length of 70 nucleotides. These fragments were mapped to RefSeq bacterial sequences using BBMap (BBTools, JGI)^4^ with a minimum identity of 85% and maximum indels of 2. Mapping files created from this step were used to mask viral matches in bacterial sequences. Masking of viral matches in addition to low complexity regions was carried out using BBMask (BBTools, JGI) with an entropoy threshold of 0.7 as suggested in BBMask Guide.^5^ The same was done for RefSeq archaeal sequences to mask matches for archaeal and bacterial viruses and prophages. All 347 virome datasets—producing 1,000 bp or longer contigs—were mapped to the RefSeq sequences (bacterial and archaeal sequences, masked for viral matches, in addition to fungal and protozoal sequences) and the proportion of bases mapping to cellular sequences was calculated.

In their assessment of the influence of cellular contamination of viromes, Roux and colleagues (Roux et al., 2013) found that the highest ratio of rDNA in 67 published viromes was 5.3‰, which was considered non-negligible extent of contamination. Therefore, we considered any virome with a ratio of rDNA greater than 5‰ to be excessively contaminated and was removed from further consideration. We also combined rDNA ratio with another strongly correlated measure, the ratio of reads mapped to potential cellular contaminants. Hence, viromes with this ratio exceeding 2% were also excluded. The remaining viromes (143) had their contigs cleaned up from cellular sequences. To clean up contigs, clean reads of each virome were mapped to corresponding contigs and those contigs showing less than 95% coverage were discarded. Dereplication of contigs using cd-hit-est v4.7 (Li and Godzik, 2006) was done to obtain a non-redundant set of contigs (options: -c 0.95 -n 10 -d 0). Generally, cd-hit keeps the longest contig when redundancy occurs. The non-redundant contigs were processed using VIBRANT (Virus Identification By iteRative ANnoTation; Kieft et al., 2020) v1.2.0, a novel tool that identifies viral contigs by combining a supervised machine learning approach with viral scoring obtained by annotations using Pfam, Virus Orthologous Groups (VOG) and Kyoto Encyclopedia of Genes and Genomes (KEGG). VIBRANT was used with the default settings.

### Viral clustering

Contigs longer than or equal to 10 kbp, identified by VIBRANT as being viral were clustered into viral operational taxonomic units (vOTUs) using cd-hit-est v4.7 (Li and Godzik, 2006) according to the guidelines suggested by Roux and colleagues (Roux et al., 2019), which suggested using 95% identity (also referred to as average nucleotide identity, ANI) and 85% coverage (also referred to as alignment fraction, AF) of the shorter sequence (options: -c 0.95 -n 10 -d 0 -G 0 – aS 0.85).

We sought to identify which of these vOTUs could belong to potentially novel viral genera/subfamilies. To this end, we downloaded Integrated Microbial Genome/Virus (IMG/VR v.2.0^6^; Paez-Espino et al., 2018) database on July 2, 2019. IMG/VR database is the largest and most comprehensive database of viral genome sequences. It was composed of 760,453 viral genomic fragments. We clustered VIBRANT vOTU representatives together with sequences from IMG/VR database into viral clusters (VCs) of genus-rank using vConTACT2 (v.0.9.19; Bin Jang et al., 2019). Since the number of sequences was huge, we had to split IMG/VR database into 39 files, adding VIBRANT vOTU representatives to each of these files and running vConTACT2, separately for each of these combinations (options: --rel-mode Diamond --db None --pcs-mode MCL --vcs-mode ClusterONE --threads 28). The 40th vConTACT2 run was similarly done, but using VIBRANT vOTU representatives alone, while vConTACT2 database was set to Refseq - release 201 (released on July 22, 2020) prokaryotic viruses (vConTACT2 option --db ProkaryoticViralRefSeq201-Merged). Generated VCs were merged by adding members of these clusters to a NetworkX (v.2.4; Hagberg et al., 2008) nondirected graph then calling upon the graph’s connected components using a custom Python script. vConTACT2 (Bin Jang et al., 2019) clusters viral genomes into viral clusters (VCs) approximately equivalent to genus level as defined by the International Committee on Taxonomy of Viruses (ICTV). vConTACT2 assigns genomes different statuses based on their clustering according to their shared gene content. Genomes can be referred to as (a) clustered, when clustering has occurred with high-confidence, (b) singleton, when they have few or no shared genes, (c) overlap when they have shared genes with genomes from more than one VC, or (d) outlier, when they have shared genes with other genomes, but not enough for high-confidence clustering into genus-level VC.

### Genome quality and completeness

Genome quality and completeness were obtained using VIBRANT v1.2.0, which relies on four different parameters for genome completeness: (a) the contig/scaffold being circular, (b) VOG annotations, (c) total VOG nucleotide replication proteins, and (d) total VOG viral hallmark proteins (Kieft et al., 2020).

### Host prediction for viral genomes

Host prediction was done using CrisprOpenDB tool, which relies on matches between viral genomes and an extended database of clustered regularly interspaced palindromic repeats (CRISPR) spacers, comprising more than 11 million spacers (Dion et al., 2021). The tool was run with a maximum number of mismatches of two. Taxonomic lineages of the predicted hosts were extracted from NCBI taxonomy (Schoch et al., 2020), with the help of ncbitax2lin tool v2.3.2 (Xue, 2022).

### Abundance of selected genomes/genome fragments

Clean reads pertaining to each freshwater biome were mapped to the selected 19,210 genomes/genome fragments longer than 10 kbp using BBMap (BBTools, JGI)^7^ with minid = 0.9, i.e., minimum identity set to 90%. Abundance was calculated based on the number of reads mapping to each genome/genome fragment and normalized to the total number of reads of each freshwater biome as well as contig length; so, relative abundance was expressed as number of reads per million reads per million bases. Intersections between biomes based on genomes/genome fragments present in each biome was calculated by combining genomes/genome fragments with nonzero abundances in each biome into a list of sets in R v.4.0.2 (R Development Core Team, 2020). Intersections between biomes based on shared genomes/genome fragments with nonzero abundances, were plotted using upset function of UpSetR package v.1.4.0 (nsets = 8, order.by = “freq,” nintersects = 50, all other arguments set to default; Conway et al., 2017). Phylogenomic analysis of the contigs with nonzero abundance among all biomes was carried out by the VICTOR web service,^8^ a method for the genome-based phylogeny and classification of prokaryotic viruses (Meier-Kolthoff and Göker, 2017). All pairwise comparisons of the nucleotide sequences were conducted using the Genome-BLAST Distance Phylogeny (GBDP) method (Meier-Kolthoff et al., 2013) under settings recommended for prokaryotic viruses (Meier-Kolthoff and Göker, 2017). The resulting intergenomic distances were used to infer a balanced minimum evolution tree with branch support *via* FASTME including subtree pruning and regrafting (SPR) postprocessing (Lefort et al., 2015) for the formula D4, which was selected because of its robustness for incomplete genomic sequences (Meier-Kolthoff and Göker, 2017). Branch support was inferred from 100 pseudo-bootstrap replicates each. The tree was rooted at the midpoint (Farris, 1972) and visualized with ggtree (Yu, 2020). Taxon boundaries at the species, genus and family levels were estimated with the OPTSIL program (Göker et al., 2009), the recommended clustering thresholds (Meier-Kolthoff and Göker, 2017) and an *F* value (fraction of links required for cluster fusion) of 0.5 (Meier-Kolthoff et al., 2014).

### Genome annotations and detection of AMGs

Genome annotation was done using VIBRANT (Kieft et al., 2020), which integrates annotations from KEGG, Pfam, and VOG databases. Detection of AMGs was also done using VIBRANT (Kieft et al., 2020), which relies on KEGG annotations in the identification of AMGs. Contigs used for detection of AMGs were the ones initially identified by VIBRANT as viral, i.e., the 273,365 contigs. AMGs annotated as the psbA photosystem II protein D1 (PsbA) were further studied to evaluate the relatedness of these AMGs with previously reported PsbA reference sequences. To this end, we collected the AMGs identified in this study as PsbA (331 sequences) as well as viral psbA reference sequences from NCBI protein database using this search query: {(psba[Gene Name]) AND viruses[Organism] AND refseq[filter]},^9^ which gave 22 sequences. Both datasets (Freshwter and RefSeq) were dereplicated using cd-hit (Li and Godzik, 2006; parameters: -c 0.95 -n 5 -d 0) to generate 161 and 12 sequences, respectively. Sequences were combined, then aligned using mafft v7.490 (Katoh and Standley, 2013; parameters: --globalpair --maxiterate 1,000). Columns with more than 50% gaps were removed using trimal v1.4 (Capella-Gutiérrez et al., 2009). The phylogenetic tree was inferred using fasttree v2.1.11 (Price et al., 2010) with default parameters. The tree was visualized using the Interactive Tree of Life (iTOL) online tool (Letunic and Bork, 2021).

### Taxonomy of viral contigs

On January 7, 2021, both taxdump and prot.accession2taxid.FULL files were downloaded from NCBI Taxonomy FTP site.^10^ These files were used to extract NCBI protein accessions belonging only to Viruses Superkingdom. These accessions were then used to extract viral protein sequences from the non-redundant protein (nr) database fasta file, downloaded from NCBI BLAST database FTP site^11^ on January 7, 2021. Viral proteins extracted from nr were used to make a custom DIAMOND (Buchfink et al., 2015) database, here referred to as viral nr, which included 3,267,301 protein sequences. Open reading frames (ORFs) were called from contigs identified by VIBRANT as viral (273,365 contigs), using prodigal v2.6.3 (Hyatt et al., 2010) in its meta mode. ORFs called from contigs were aligned to viral nr using DIAMOND (Buchfink et al., 2015; Command: blastp; Options: --top 50 --matrix BLOSUM62 --evalue 0.001 --block-size 2 --index-chunks 4 --tmpdir.). Taxonomoy for each contig was identified using CAT v5.2.1 (von Meijenfeldt et al., 2019), which relies on a voting-based method that considers all ORFs of a particular contig and assigns taxonomy to a contig based on the lowest common ancestor, accordingly. Taxonomy assigned to contigs based on less than 50% of ORFs was disregarded. Additionally, during counting of contigs assigned to different taxonomic ranks, taxonomic assignments marked with an asterisk (a feature of CAT to mark suggestive taxonomic assignments, where conflict between classifications was not enough to allow more confident classification), were also removed.

## Results

### Dataset overview

#### Quality control and sequence assembly

We downloaded 380 freshwater viral metagenomes from SRA samples from several countries/locations throughout the globe, encompassing four out of seven continents (Supplementary Table S2, Figure S2). These viromes were collected from diverse freshwater biomes, e.g., lakes, groundwater, wastewater, etc. (Table 1). Raw sequences of these 380 metagenomes amounted to over 614 gigabases (1.28 terabytes). Only 476 gigabases (0.96 terabytes) passed quality control (Supplementary Table S3). Out of 380 metagenomes, 347 (91.3%) generated contigs longer than 1,000 bp and those were the ones used for further study. The total number of contigs longer than 1,000 bp was 3,697,298 (Supplementary Table S4).

**Table 1:** Freshwater biomes of the metagenomes used in this study.

	Number of metagenomes	
Freshwater biome	Before data analysis	After data analysis
Ballast water	5	–
Catchment	18	–
Estuary	16	10
Fish pond	3	3
Groundwater	8	8
Harbor water	3	–
Lake	84	37
Pure water (MilliQ)	26	–
Reclaimed water	6	3
Water reservoir	26	4
Watershed	182	75
Wastewater treatment plant	3	3
Total	380	143

#### Assessment and removal of cellular contamination

We carried out several steps to assess and remove cellular contamination (Supplementary Figure S1). Only two metagenomes (SRR1658890 and SRR1658891) showed no detected ribosomal DNA sequences or any reads mapped to possible cellular contaminants (Supplementary Table S5). Generally, the extent of detected ribosomal DNA sequences and level of reads mapped to possible cellular contaminants, those two measures of cellular contamination, agreed remarkably with strong correlation (Supplementary Figure S3). For six metagenomes out of 347 (0.9%), percent bases mapped to cellular sequences exceeded 90%, attributed mainly to ribosomal DNA sequences (Supplementary Table S5). We decided to proceed only with metagenomes with no more than 2% bases mapped to cellular sequences and less than 5‰ predicted rDNA bases. Only 143 viral metagenomes out of 347 (41%) fulfilled these criteria. Out of these 143 metagenomes, 10 (7%) showed no predicted rDNA, 80 (56%) showed less than 0.2‰ rDNA and 53 (37%) showed more than 0.2‰ rDNA and up to less than 5‰. The total number of contigs for the selected 143 viromes was 1,778,319. After the removal of contigs showing less than 95% coverage with clean reads, the number of contigs was reduced to 1,763,349. Then, after dereplication and elimination of likely redundant contigs, the number of contigs became 1,226,122 (≥1,000 bp). VIBRANT (Kieft et al., 2020) was able to predict 273,365 contigs out of this non-redundant contig pool to belong to phages.

#### Viral clusters

**Figure 1:**
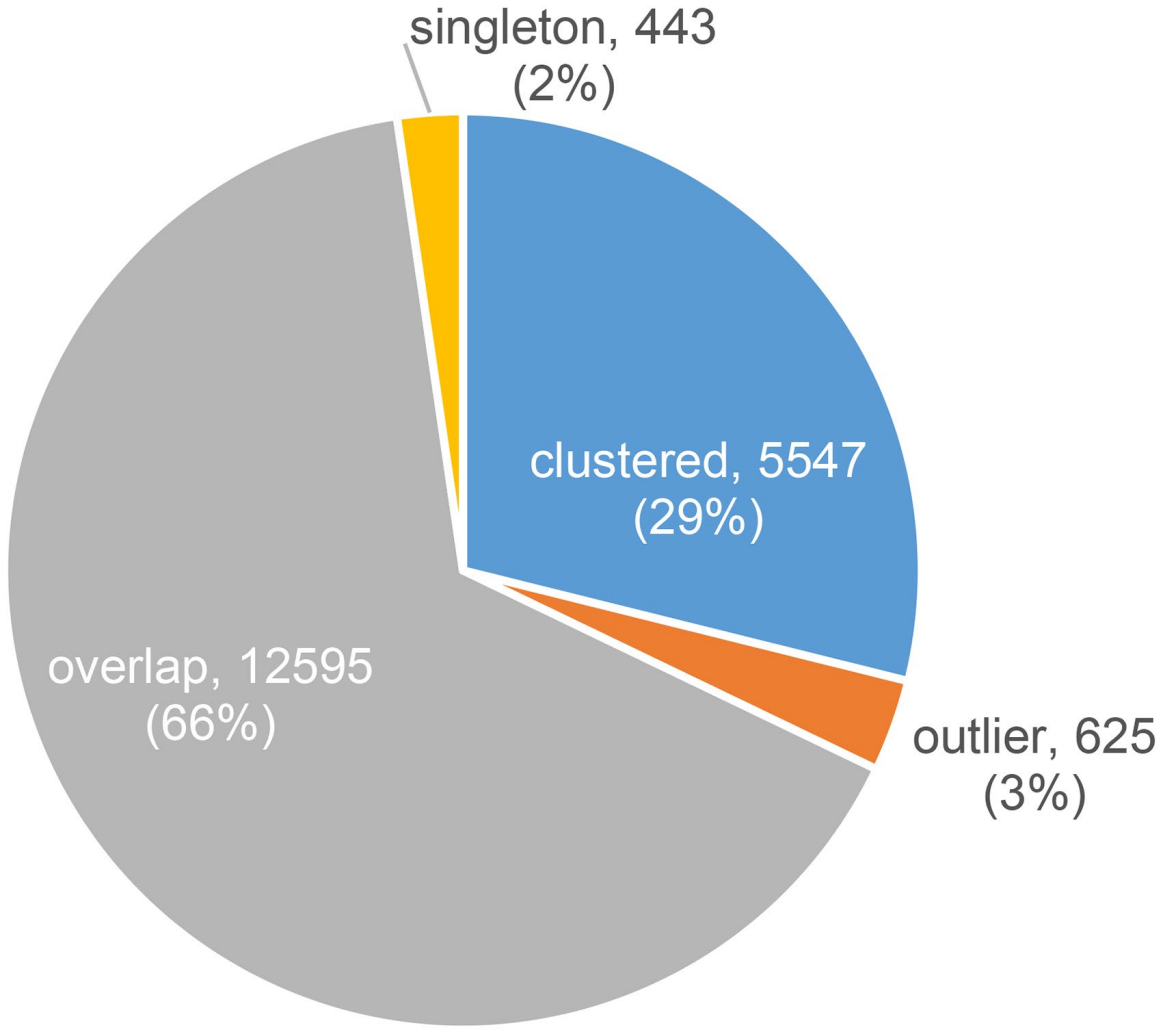
Distribution of contigs according to their clustering result based on their shared proteins using vConTACT2.

Contigs predicted to be viral that were longer than or equal to 10,000 bp (20,107 contigs) were clustered into vOTUs. vOTUs representatives (19,210 contigs) were clustered based on their shared proteins together with JGI IMG/VR sequences as well as RefSeq prokaryotic viruses release 201. Out of 19,210 contigs 5,547 (28.9%) were clustered into clusters of two or more members (Figure 1). The total number of these clusters was 406. Out of these 406 clusters, only 31 clusters (7.6%), were solely composed of contigs from the studied freshwater metagenomes (totaling 138 contigs) i.e., these 31 clusters did not include any sequence from the JGI IMG/VR or RefSeq prokaryotic viruses. Less contigs were identified as singletons or outliers (~2 and ~ 3%, respectively; Figure 1). Most of the contigs, however, were denoted as overlaps (~66%; Figure 1) meaning that they were ascribed to more than one cluster, obscuring their cluster affiliation.

We considered contigs, which did not cluster with any previously known sequences, as novel genus-rank contigs. These included clustered contigs that did not include in their clusters any sequence from the JGI IMG/VR or RefSeq prokaryotic viruses, in addition to singletons and outliers, amounting to 1,206 contigs (6%; Table 2).

**Table 2:** Genome quality versus clustering status.

		Genome quality				Total
		Complete circular	High quality draft	Medium quality draft	Low quality draft	
Clustering status	Clustered, but not with any previously known sequence	1	7	14	116	138
	Clustered with previously known sequence(s)	23	66	603	4,717	5,409
	Singleton	–	1	15	427	443
	Outlier	–	1	23	601	625
	Overlap	70	380	1798	10,347	12,595
	Total	94	455	2,453	16,208	19,210

### Recovered viral genomes

Out of the 19,210 vOTUs representatives, 549 complete and near-complete (high quality draft) genomes could be recovered. Out of these 549 genomes, novel genus-rank genomes, as defined above, were 10 (one complete and nine high quality draft genomes, Table 2). For these novel genomes, genome size ranged between 15,100 bp and 156,997 bp (Mean = 48,712.6 bp, Median = 34,324.5 bp). Coding sequences (CDS) were 561, 237 (42.2%) of which were completely unknown, i.e., had no hits in any of the KEGG, Pfam, or VOG databases. Moreover, 82 CDS (14.6%), despite having VOG hits, the proteins were of unknown function, hence designated hypothetical proteins. The remaining CDS (242 gene product, 43.1%) were largely dominated by proteins involved in DNA binding, replication, recombination, repair and metabolism in addition to phage structural proteins (Supplementary Table S6). As for the full record of viral genomes/genome fragments (273,365 contigs), out of 2,119,106 CDS, only 429,341 (20.2%) could be functionally annotated. For a complete list of annotations of all recovered genomes/genome fragments, please refer to Data Availability Section.

### Predicted hosts of viral genomes

Out of 19,210 viral genomes, hosts could be successfully predicted for only 343 genomes (1.79%). All predicted hosts belonged to Bacteria (Supplementary Table S7). These hosts are members of nine different bacterial phyla, the most abundant of which were Proteobacteria (222 genomes), followed by Bacteroidetes (52 genomes) and Actinobacteria (34 genomes). The total number of detected genera was 141, with the most abundant genus being Flavobacterium (23 genomes), followed by Pectobacterium (19 genomes) and Salinispora (14 genomes).

### Abundance of vOTUs representatives in the studied biomes

**Figure 2:**
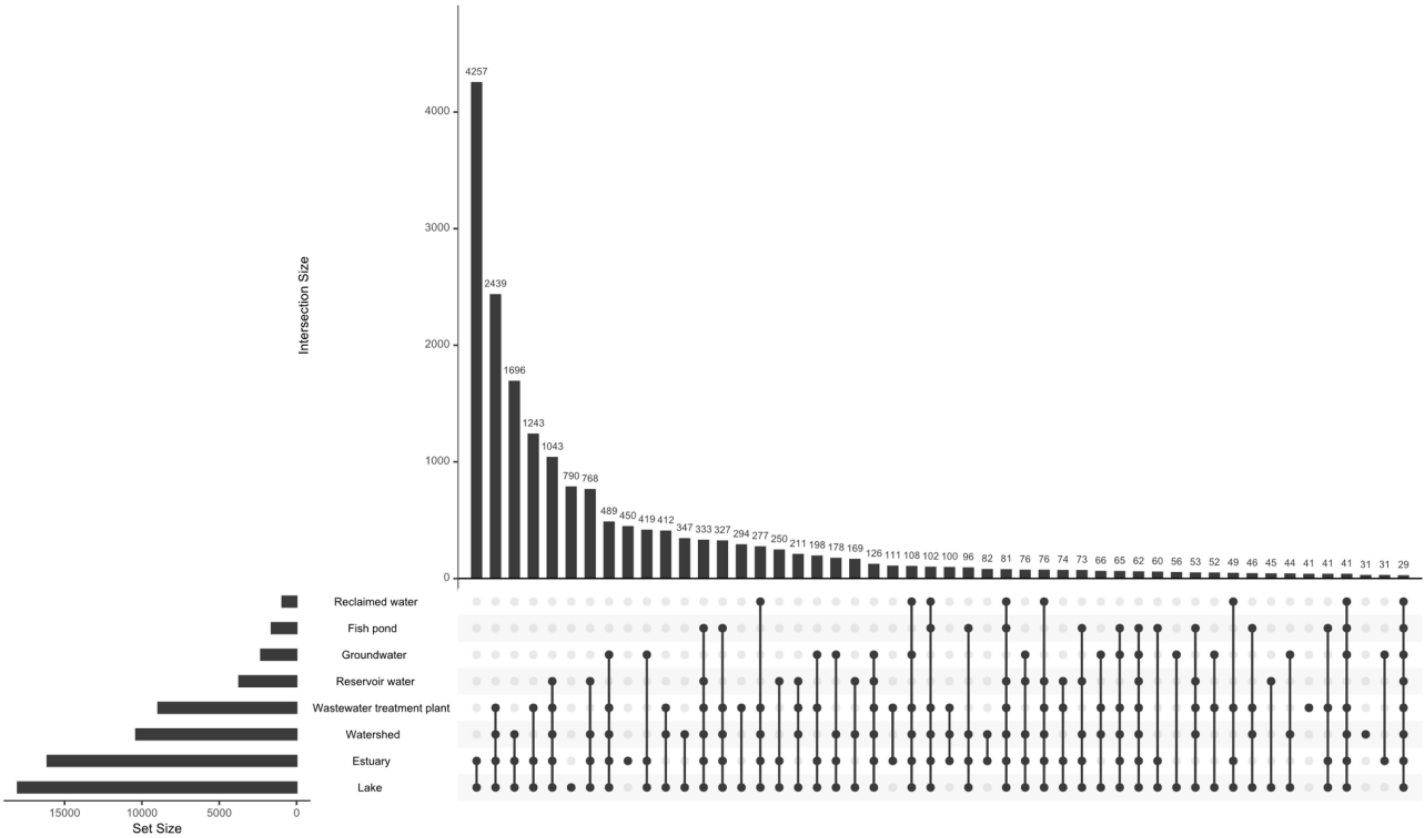
Intersection between different biomes based on common contigs with nonzero abundance. The figure was generated using upset function of UpSetR package v.1.4.0 (nsets = 8, order.by = “freq,” nintersects = 50, all other arguments set to default; Conway et al., 2017).

**Figure 3:**
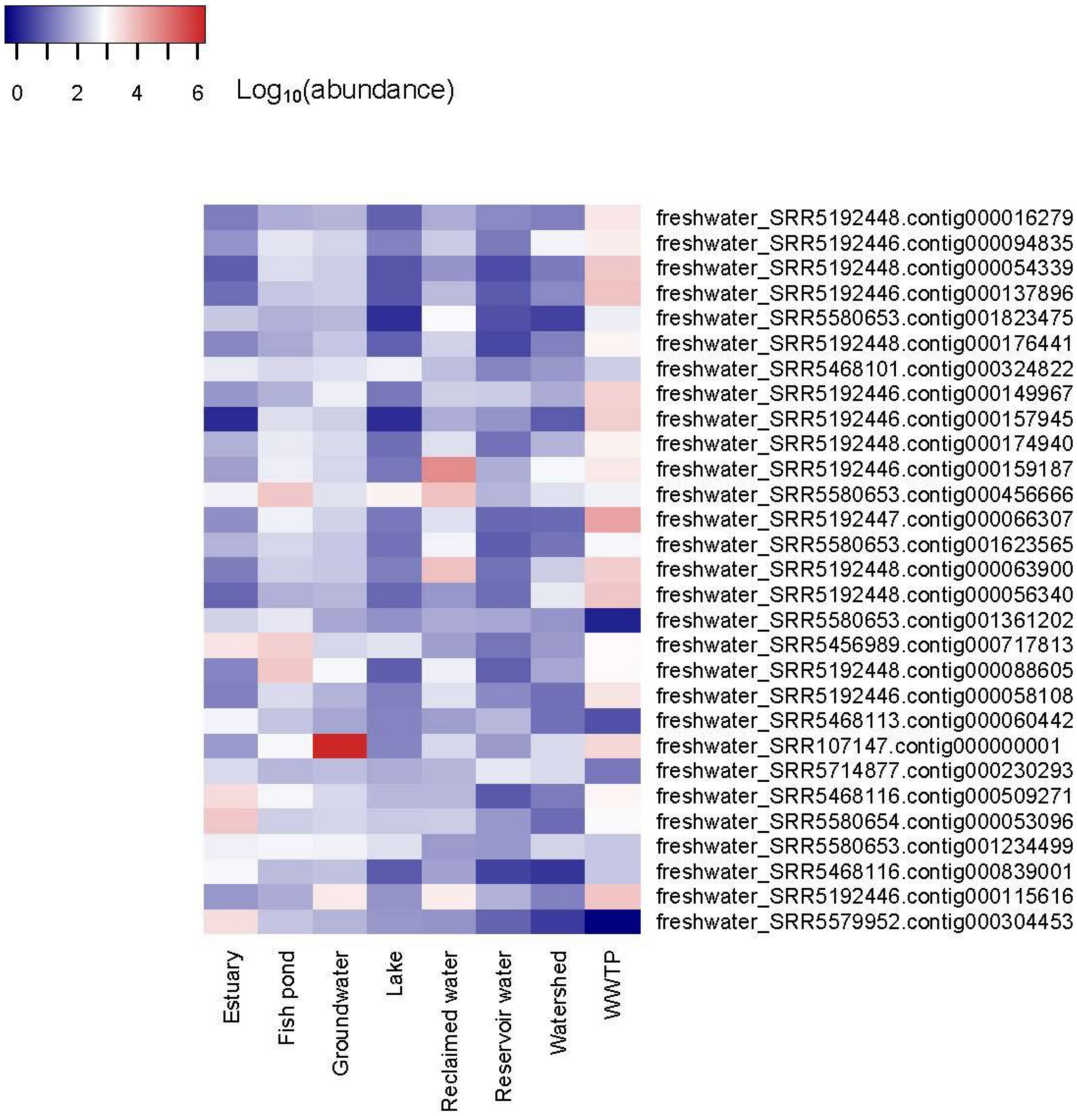
Heatmap of the abundance of 29 contigs with nonzero abundance among all studied biomes. The heatmap was generated using Heatmap3 R package v.1.1.7 (Zhao et al., 2014) in R v.4.0.2 (R Development Core Team, 2020) after log_10_ transformation of abundance values for better visualization.

**Figure 4:**
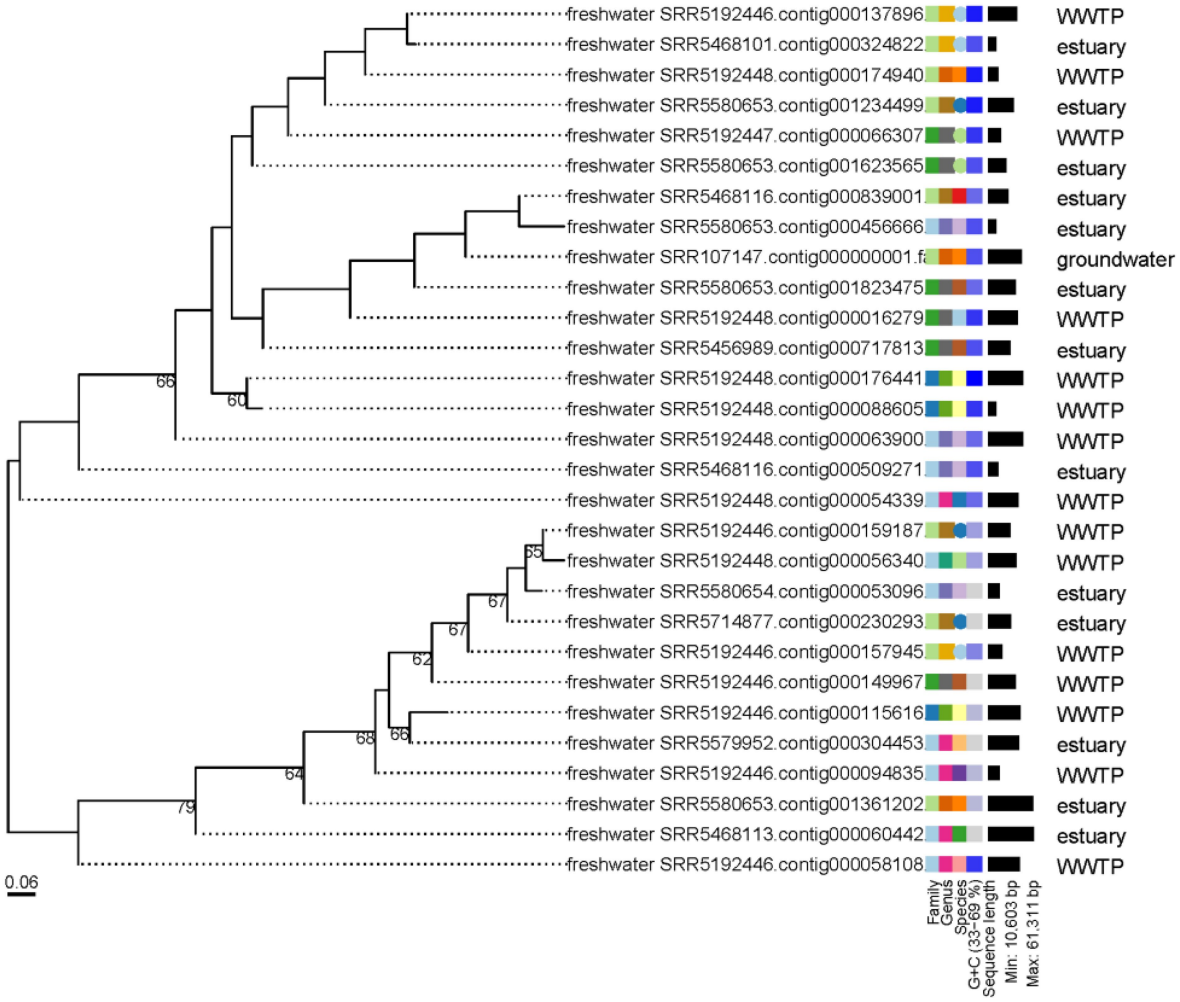
Phylogenomic tree of the 29 contigs with nonzero abundance among all studied biomes. The numbers above branches are GBDP pseudo-bootstrap support values from 100 replications. The branch lengths of the resulting VICTOR tree are scaled in terms of the D4 distance formula used.

The abundance of the 19,210 vOTUs representatives was calculated for each biome and intersections between biomes based on common contigs with nonzero abundance among different biomes was determined (Figure 2). Only 29 contigs (< 0.2%) were found to have nonzero abundance in all studied biomes. A list of all common contigs among different biomes is included in Supplementary Table S8. The abundance of the 29 contigs common among all studied biomes is shown in Figure 3. It is worth noting that these 29 contigs originally belonged to only three biomes, namely estuary, groundwater and WWTP (Figure 4). Figure 4 shows the phylogenomic tree for these 29 contigs, yielding an average support of 53%. Although these contigs could be clustered into two major clades (Figure 4), no particular pattern could be observed concerning the clustering of the contigs based on the biomes they originated from. The OPTSIL clustering yielded 15 species clusters, eight genus clusters and four family level clusters.

**Figure 5:**
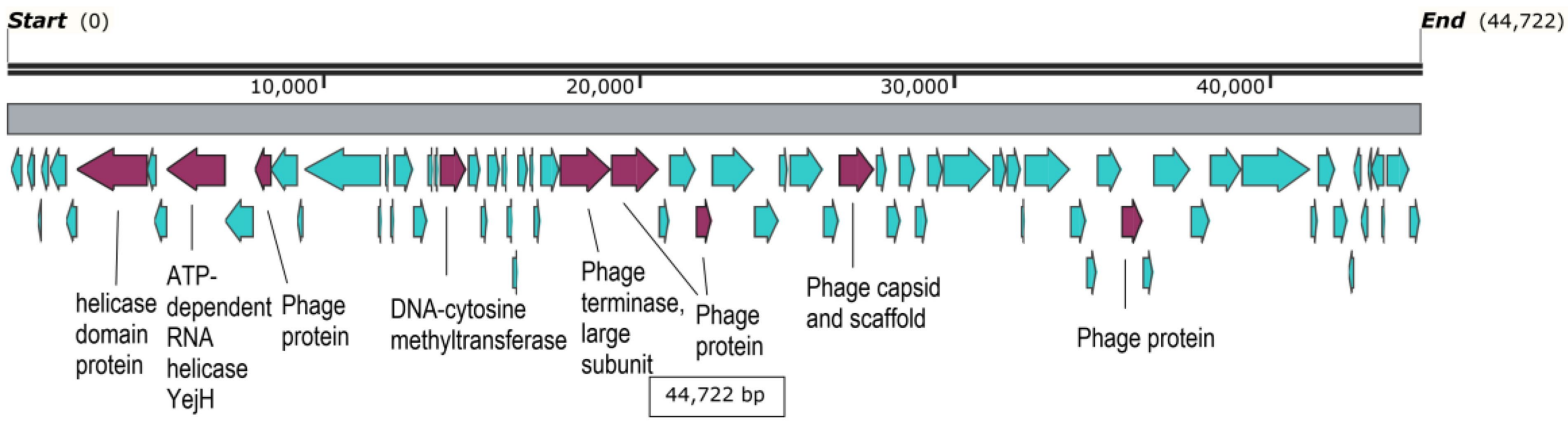
Gene map of freshwater_SRR107147.contig000000001. CDS highlighted in cyan are hypothetical proteins, while the purple ones have labeled annotations.

Interestingly, one contig, namely freshwater_SRR107147.contig000000001 showed the highest abundance among all abundance values (Supplementary Table S9; Figure 3). Its abundance was highest in groundwater, comprising more than 7% of all groundwater reads or a relative abundance of 1.6 × 10^6^ reads per million reads per million bases. It’s worth noting that this contig was classified as a medium-quality draft genome by VIBRANT. The genome size was 44,722 bp. While most of its coding sequences (CDS) were identified as hypothetical proteins, only nine out of 74 CDS (12.2%) were annotated to several functions, including helicases, DNA-cytosine methyltransferase, phage terminase, as well as capsid protein and other uncharacterized phage proteins (Figure 5). Taxonomic annotation of this genome showed that it belongs to *Caudovirales*, which means that it is a double-stranded DNA (dsDNA) phage. Host prediction for this genome showed that its potential host is *Methylomonas* (Gammaproteobacterium).

### Auxiliary metabolic genes

A total of 17,139 AMGs (Supplementary Table S10) were detected in the contigs deemed by VIBRANT as viral (273,365 contigs). These AMGs belonged to 11 different categories of metabolism (Supplementary Figure S4, Table S11). Top three metabolic pathways with the highest numbers of AMGs pertained to carbohydrate metabolism with a total of 5,885 AMGs, followed by amino acid metabolism (4,966 AMGs), then metabolism of cofactors and vitamins (4,675 AMGs). Bottom three metabolic pathways, with the lowest numbers of AMGs, belonged to lipid metabolism, metabolism of other amino acids and xenobiotics biodegradation and metabolism with AMGs counts of 203, 167 and 71, respectively. AMGs could be detected in all studied biomes except fish ponds (Supplementary Table S10). Most of these AMGs were detected in estuary samples [15,484 out of 17,139 (90.3%)], followed by 1,282 AMGs in WWTP (7.5%). Diversity of the detected AMGs with regards to the pathways they belonged to was highest in estuary and WWTP samples (11 pathways, each), but was still relatively high in reservoir and lake samples (nine and eight pathways, respectively), despite the limited numbers of AMGs in these biomes. Numbers of detected AMGs and the pathways they belonged to were lowest in groundwater, reclaimed water and watershed (six, four and three AMGs and two, two and three pathways, respectively). Concerning the taxonomy of these AMGs, it could be assigned at the order level for 12,240 AMGs out of 17,139 (71.4%; Supplementary Table S10). More than 12,000 of these AMGs (98.7%) belonged to the Order *Caudovirales*. On the family level, only 2,882 out of 17,139 AMGs (16.8%) could be assigned taxonomy. Out of these 2,882 AMGs, 1,488 AMGs (51.6%) belonged to *Myoviridae*.

Additionally, manual inspection of individual AMGs (Supplementary Table S10) highlighted the presence of putative antibiotic resistance proteins e.g., CDS annotated as beta-lactamase superfamily domain (five CDS), one CDS annotated as metallo-beta-lactamase superfamily and another annotated as cephalosporin hydroxylase. Similarly, 265 CDS were annotated as photosystem II protein D1 (PsbA) and 93 CDS annotated as photosystem II protein D2 (PsbD), involved in photosynthesis. Moreover, several CDS involved in xenobiotic degradation, particularly hydrocarbon degradation were detected e.g., MhpE (5 CDS) involved in the degradation of aromatic compounds, and components of the phenylacetate degradation pathway, which are also involved in the degradation of aromatic compounds e.g., PaaA (one CDS), PaaD (four CDS) and PaaK (two CDS).

**Figure 6:**
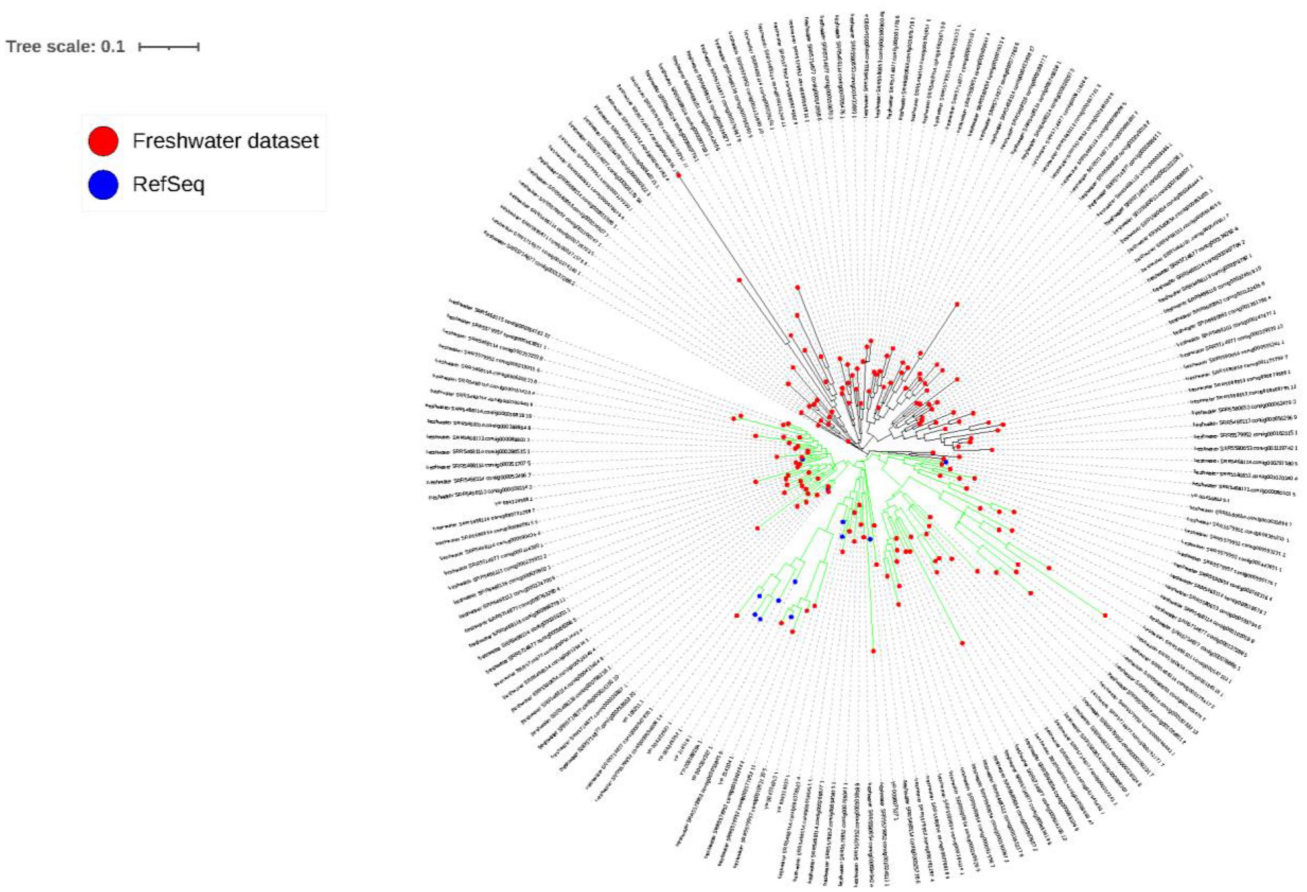
PsbA phylogenetic tree. Red circles at the tip of branches denote sequences identified in the current study, while blue circles denote RefSeq PsbA sequences collected from NCBI. Two main clades can be observed, whose branches are colored in green and black.

The PsbA phylogenetic tree (Figure 6) reveals the clustering of sequences into two main clades (green and black branches), with 96 and 78 sequences, respectively. The green-branched clade contains all of the NCBI’s RefSeq PsbA sequences (12 sequences), with particular concentration of most of these sequences along the middle subclade of the main, green-branched clade. On the other hand, the black-branched is solely made from sequences from the currently studied dataset (78 out of 162 dereplicated sequences (48.1%)).

### Taxonomy of viral contigs

ORFs called from VIBRANT-identified viral contigs were aligned to viral nr and taxonomy was inferred using CAT v5.2.1 (von Meijenfeldt et al., 2019). Out of 273,365 contigs, 173,392 contigs (63%) could be classified as Viruses at the Superkingdom rank (Table 3) with at least 50% of ORFs supporting this classification. Decreasing number of contigs could be assigned to lower ranks with 43, 30, 30 and 15% of contigs assigned to known viral phyla, classes, orders and families, respectively. Uroviricota was the phylum with the highest number of assigned contigs, amounting to 116,250 (~43% of viral contigs). On the class and order levels, most assigned contigs pertained to Caudoviricetes and *Caudovirales*, respectively with exactly the same number of contigs (80,850 contigs). As for the family level, three different families, namely *Siphoviridae* (16,858 contigs), *Myoviridae* (12,840 contigs) and *Podoviridae* (7,449 contigs) recruited the highest number of assigned contigs compared to the rest of assigned families. Detailed taxonomy of all assigned contigs can be found in Supplementary Table S12.

**Table 3:** Breakdown of the different taxonomic assignments of viral contigs.

Classification	Breakdown		Total	Percentage
Superkingdom	Viruses	173,392	173,392	63%
Phylum	Cressdnaviricota	90	118,471	43%
	Hofneiviricota	1		
	Nucleocytoviricota	661		
	Peploviricota	1		
	Phixviricota	1,209		
	Preplasmiviricota	259		
	Uroviricota	116,250		
Class	Arfiviricetes	13	83,030	30%
	Caudoviricetes	80,850		
	Faserviricetes	1		
	Malgrandaviricetes	1,209		
	Maveriviricetes	258		
	Megaviricetes	660		
	Repensiviricetes	38		
	Tectiliviricetes	1		
Order	Algavirales	461	82,937	30%
	Caudovirales	80,850		
	Cirlivirales	13		
	Geplafuvirales	38		
	Imitervirales	94		
	Petitvirales	1,209		
	Pimascovirales	31		
	Priklausovirales	239		
	Tubulavirales	1		
	Vinavirales	1		
Family	Ackermannviridae	24	41,027	15%
	Adintoviridae	15		
	Autographiviridae	1754		
	Baculoviridae	1		
	Chaseviridae	8		
	Circoviridae	12		
	Corticoviridae	1		
	Cruciviridae	10		
	Demerecviridae	2		
	Drexlerviridae	4		
	Fuselloviridae	1		
	Genomoviridae	38		
	Herelleviridae	28		
	Inoviridae	1		
	Iridoviridae	27		
	Lavidaviridae	239		
	Marseilleviridae	2		
	Microviridae	1,209		
	Mimiviridae	81		
	Myoviridae	12,840		
	Phycodnaviridae	413		
	Pithoviridae	10		
	Podoviridae	7,449		
	Siphoviridae	16,858		

## Discussion

In this study, we sought to investigate freshwater viromes in a global context, especially given the little knowledge available about viromes in this environment. A simple PubMed search with search queries: (marine viral metagenomes) versus (freshwater viral metagenomes) shows an annual number of publications more than two times higher for marine viral metagenomes compared with freshwater viral metagenomes (Supplementary Figure S5). We downloaded 380 freshwater viral metagenomes from SRA amounting to more than one terabyte of data. We set out to leverage this big data to obtain valuable information about viruses in freshwater. In order to do that, we had to carefully consider cellular contamination in the downloaded datasets. Surprisingly, the rDNA content of many datasets approached or even exceeded 90% of the total number of reads (Supplementary Figure S2; Zolfo et al., 2019). This strikingly high rDNA content most likely means that these datasets were falsely labeled as viral metagenomes, while they are in fact rDNA amplicon sequences. It is not uncommon for viral metagenomic projects to sequence 16S rDNA amplicons to correlate viral with bacterial diversity (Parsley et al., 2010; Park et al., 2011; Skvortsov et al., 2016). Yet, the mislabeling of these amplicon sequences as viral metagenomes in metadata warrants attention to information submitted with each file in databases to avoid unnecessary processing of such files and waste of time and computational resources.

To look for novel genomes, we clustered VIBRANT viral contigs greater than 10 kb into vOTUs then using vConTACT2 (Bin Jang et al., 2019), we could cluster these contigs based on their shared proteins with JGI IMG/VR (Paez-Espino et al., 2018) and/or RefSeq prokaryotic viral genomes. Novel genomes/genome fragments, based on this approach, were 1,206, only 10 of which were complete, circular or high-quality draft genomes. The number might seem small, but it is still considerable, given the fact that clustering was done with the most comprehensive and diverse available viral resource JGI IMG/VR (Paez-Espino et al., 2018). The percentage of functionally annotated CDS of these 10 complete/high-quality draft genomes was much higher than the overall percentage of functionally annotated CDS of all viral contigs (43.1% versus 20.2%). This could possibly be explained given that VIBRANT’s ability to identify genome completeness relies on the identification of viral hall mark proteins, which could also mean that complete and high-quality draft genomes would have better levels of annotation. We have previously reported similar annotation ratio (21.2%) when Pfam was used to annotate human virome protein clusters (Elbehery et al., 2018). Interestingly, Pfam is also used in VIBRANT’s annotation of viral contigs, in addition to KEGG and VOG (Kieft et al., 2020). Generally, the fact that viruses are underrepresented in databases reduces the level of functional annotation of metagenomic sequences. For example, NCBI Genome resource^12^ contains only 41,909 viral genomes versus 298,742 prokaryotic genomes, as of January 16, 2021.

Exploring the abundance of vOTUs showed that less than 0.2% of these contigs had nonzero abundance in all of the studied biomes. This could potentially mean that the core (shared) freshwater virome is minimal and that each freshwater environment has a distinct virome, which is site-specific. This explanation agrees with previous studies which highlighted that virome diversity differs among samples of the same environment, including those spatially close to each other. These studies suggest that viral diversity is mainly dictated by environmental variables specific to each sample site (Saxton et al., 2016; Adriaenssens et al., 2017). Looking up the taxonomy of the contigs with nonzero abundance in all studied biomes in Supplementary Table S12 shows that 41.4% of them could not be assigned taxonomy, while the rest mostly belonged to *Caudovirales* with no enough support to identify taxonomy at the family level. Phylogenomics and estimation of taxonomic boundaries of these contigs showed that they are composed of limited numbers of viral species, genera and families (Figure 4). This observation could denote a limited complexity of the freshwater core virome as previously suggested for gut and ocean viromes (Brum et al., 2015; Broecker et al., 2017).

The most abundant genome with nonzero abundance in all biomes was freshwater_SRR107147.contig000000001. The functional annotation of this genome showed several CDS with different functions, including helicases, which are important for genome replication (Frick and Lam, 2006). DNA-cytosine methyltransferase was another protein detected in this genome, whose function is to protect bacteriophages against restriction-modification in bacterial hosts (Murphy et al., 2013). Ultimately, phage terminase is another detected protein of particular importance in genome packaging at the end of the lytic cycles of infection (Shen et al., 2012). It is worth noting that *Methanococcus*, the potential host of this genome is usually isolated from freshwater habitats and that methanotrophs in general constitute a large fraction of the microbial diversity in many freshwater aquatic environments (Bowman, 2006). This probably explains why freshwater_SRR107147.contig000000001 phage genome was most abundant in a multitude of freshwater environments.

Auxiliary metabolic genes is a term that has first been suggested by Breitbart and colleagues (Breitbart et al., 2007) to describe genes of bacterial host origin believed to be leftovers of previous horizontal gene transfer processes, found in bacteriophages and play important role in viral infection of bacteria. Actually, bacteriophages have been thought to utilize such genes in an integrative way to enhance infection efficiency. The variation in the number and diversity of detected AMGs among different biomes (Supplementary Table S10) could be interpreted on the merits of the differences in sequencing depth as well as the variation in environmental variables among different biomes, which warrants the presence of diverse AMGs to maximize host fitness in the face of different conditions (Warwick-Dugdale et al., 2019b). Notably, The highest numbers of AMGs were detected in the *Myoviridae* family (Supplementary Table S10), which was recently reported (Heyerhoff et al., 2022), probably indicating that phages of this particular family have special emphasis on the employment of AMGs in their life cycles potentially for an overall improved viral fitness. In this study, numerous AMGs, belonging to several metabolic pathways could be detected. For example, we detected photosynthetic core photosystem II reaction center proteins PsbA and PsbD. These proteins are widespread in cyanophages in both marine (Sullivan et al., 2006) and freshwater (Ruiz-Perez et al., 2019) aquatic systems. During their infection of cyanobacteria, cyanophages direct cyanobacteria to express these viral proteins to enhance photosynthesis and increase ATP production (Breitbart et al., 2007; Thompson et al., 2011). Similarly, we detected more AMGs belonging to carbohydrate metabolism, particularly pentose phosphate pathway, and nucleotide synthesis (Supplementary Table S11). In fact, cyanophages make use of these AMGs to eventually increase nucleotide production, which in turn increases viral replication and production (Breitbart et al., 2007; Thompson et al., 2011). Remarkably, almost half of the PsbA AMGs detected in the currently studied freshwater dataset did not cluster with any of the PsbA reference sequences included in the constructed phylogenetic tree (Figure 6), denoting their uniqueness.

In addition to AMGs, which potentially improve infection efficiency, we detected other AMGs, such as potential antibiotic resistance proteins as well as xenobiotic degradation proteins. Antibiotic resistance genes have previously been reported in several viromes, including aquaculture wastewater (Colombo et al., 2016), river (Colombo et al., 2017), and urban surface freshwater (Moon et al., 2020). Yet, there are concerns that the reported genes are overestimated; in addition, their functionality and ability to confer resistance in bacteria are questionable (Enault et al., 2017). Therefore, experimental verification of such genes is essential. On the other hand, we could detect MhpE, PaaA, PaaD and PaaK, all of which are involved in aromatic compound degradation, which could potentially be used in bioremediation. Generally, bacteriophages could have several roles in polluted environments. The presence of environmental pollutants could induce prophages in lysogenic bacteria, increasing the overall abundance of free viruses (Cochran et al., 1998). Besides, it was shown that bacteriophages control the abundances of microbial populations, following “killing the winner” pattern with a stronger influence in diesel-contaminated water systems compared to control ones (Sauret et al., 2015). Another study (Paterson et al., 2019) suggested another model in trichloroethene-contaminated groundwater, where viruses shift from lytic to lysogenic life cycle when bacterial hosts are persistently available in low abundances. The model was called Piggyback-the-Persistent (PtP). Furthermore, bacteriophages could harbor hydrocarbon-degrading genes (Costeira, 2019). MhpE is an aldolase, constituting a part of the 3-hydroxyphenylpropionic acid degradation pathway, where it catalyzes the cleavage of 4-hydroxy-2-ketopentanoic acid, giving pyruvate and acetaldehyde, which is then converted to acetyl coenzyme A by MhpF (Diaz et al., 2001). In contrast, PaaA, PaaD and PaaK are components of the phenylacetic acid degradation pathway, where PaaK (phenylacetate-CoA ligase) catalyzes the first step in the pathway, converting phenylacetic acid to phenylacetyl-CoA, then, PaaABCDE complex catalyzes the epoxidation of the aromatic ring (Ismail and Gescher, 2012).

Taxonomic analysis of the studied viral contigs showed that most of the assigned contigs belonged to tailed phages of the order *Caudovirales*, particularly of the families *Siphoviridae*, *Myoviridae* and *Podviridae*. This taxonomy agrees with many of the reported taxonomies of many freshwater viromes (Mohiuddin and Schellhorn, 2015; Potapov et al., 2019; Rusiñol et al., 2020; Moon et al., 2020), whose most abundant taxa also belonged to *Caudovirales*, but probably with some shuffling of the order of the most abundant family among the top three, namely *Siphoviridae*, *Myoviridae* and *Podviridae*. *Caudovirales* are tailed bacteriophages with their structures made up of icosahedral head, neck and tail (Iwasaki et al., 2018). The families of this order differ mainly in tail characteristics: *Syphoviridae* with long non-contractile tails, *Myoviridae* with long contractile tails and *Podoviridae* with short tails. *Caudovirales* generally dominate databases, which could lead to some bias in taxonomic annotation (Palermo et al., 2021). Although most metagenomic studies in aquatic environments report *Caudovirales* (tailed phages) as most abundant, a global study based on electron microscopy revealed that non-tailed viruses were most abundant in marine environments (Brum et al., 2013). A similar study for freshwater is necessary.

Taken together, we have assessed freshwater viromes from several locations all around the world, giving a global highlight of viruses in this environment. Besides, we shed light on common mislabeling and unorganized metadata in databases that could lead to profoundly misled studies, or at least waste of time, effort and computational resources. We strongly recommend the implementation of a quick pipeline through which a small subset of randomly picked sequence reads from metagenomic data should run before uploading to SRA and similar repositories. The pipeline should automatically predict whether these datasets are amplicons (e.g., 16S data), microbiomes or viromes, to verify the claims of the uploaders. We also could recover many complete and high-quality draft genomes, although only a few of them could be considered novel. Moreover, our study emphasizes the role of viral AMGs in bacterial metabolism. Finally, our carefully decontaminated and cautiously selected set of viral contigs remains a potentially useful resource for future studies in environmental viromics.

## Data availability statement

All relevant data including sequences of the viral contigs, annotations, clusters and the custom Python script are available at: https://osf.io/ucqv4/ (DOI: 10.17605/OSF.IO/UCQV4).

## Author contributions

All authors listed have made a substantial, direct, and intellectual contribution to the work and approved it for publication.

## Funding

This work was funded by the German Research Foundation (DFG Emmy Noether Program, project no. 273124240 and DE2360/1–1, as well as no. 391644373 and DE2360/2–1 awarded to LD).

## Conflict of interest

The authors declare that the research was conducted in the absence of any commercial or financial relationships that could be construed as a potential conflict of interest.

## Publisher’s note

All claims expressed in this article are solely those of the authors and do not necessarily represent those of their affiliated organizations, or those of the publisher, the editors and the reviewers. Any product that may be evaluated in this article, or claim that may be made by its manufacturer, is not guaranteed or endorsed by the publisher.

## Footnotes

^1^ https://www.ncbi.nlm.nih.gov/sra

^2^ ftp://ftp.ncbi.nlm.nih.gov/refseq/

^3^ https://jgi.doe.gov/data-and-tools/bbtools/

^4^ https://sourceforge.net/projects/bbmap/

^5^ https://jgi.doe.gov/data-and-tools/software-tools/bbtools/bb-tools-user-guide/bbmask-guide/

^6^ https://genome.jgi.doe.gov/portal/IMG_VR/IMG_VR.home.html

^7^ https://sourceforge.net/projects/bbmap/

^8^ https://victor.dsmz.de

^9^ https://www.ncbi.nlm.nih.gov/protein/

^10^ ftp://ftp.ncbi.nih.gov/pub/taxonomy/

^11^ https://ftp.ncbi.nlm.nih.gov/blast/db/FASTA/

^12^ https://www.ncbi.nlm.nih.gov/genome/

## Supplementary material

SUPPLEMENTARY TABLE S1 Downloaded viral metagenomes.

SUPPLEMENTARY TABLE S2 Number of metagenomes before and after decontamination.

SUPPLEMENTARY TABLE S3 Raw and quality controlled read statistics.

SUPPLEMENTARY TABLE S4 Contig statistics.

SUPPLEMENTARY TABLE S5 Percent bases mapped to cellular sequences versus rRNA reads per 1000 reads.

SUPPLEMENTARY TABLE S6 Genbank table of the 10 novel complete and high-quality draft genomes.

SUPPLEMENTARY TABLE S7 Hosts of all 19210 vOTUs.

SUPPLEMENTARY TABLE S8 List of common contigs of non-zero abundance among different biomes.

SUPPLEMENTARY TABLE S9 Abundance of all 19210 contigs (vOTUs representatives) in all studied freshwater biomes.

SUPPLEMENTARY TABLE S10 AMG individual proteins and breakdown of their counts according to biomes and taxonomy.

SUPPLEMENTARY TABLE S11 AMG pathways.

SUPPLEMENTARY TABLE S12 Taxonomy of viral contigs.

SUPPLEMENTARY FIGURE S1 Diagram explaining how cellular contamination in the metagenomic datasets was assessed and removed.

SUPPLEMENTARY FIGURE S2 World map showing the locations of the studied 143 metagenomes. The size of the circles at each location is proportional to the number of metagenomes collected from this location.

SUPPLEMENTARY FIGURE S3 Percent bases mapped to cellular sequences versus rRNA reads per 1000 reads.

SUPPLEMENTARY FIGURE S4 Numbers of detected AMGs classified according to metabolism categories.

SUPPLEMENTARY FIGURE S5 Annual number of publications for marine viral metagenomes versus freshwater viral metagenomes.
