# Supplementary material for: Insights into the global freshwater virome
Source: Front Microbiol. 2022 Sep 28;13:953500. doi: 10.3389/fmicb.2022.953500 (PMC9554406; doi:10.3389/fmicb.2022.953500)
Supplement: SUPPLEMENTARY TABLE S2 — Number of metagenomes before and after decontamination. [file Table_2.pdf]

Table S2. Number of metagenomes used in this study before and after data analysis, and their distribution per country/location. Metagenomes were excluded if they did not fulfill the criteria outlined in Materials and Methods.

	**Number of Metagenomes**	
**Country/Location**	**Before contamination assessment**	**After contamination assessment**
**Antarctica**	31	27
**Canada**	208	75
**China**	3	3
**France**	2	1
**Norway**	10	7
**Singapore**	38	4
**Taiwan**	6	-
**UK**	1	1
**USA**	81	25
**Total**	380	143
