# Supplementary material for: Insights into the global freshwater virome
Source: Front Microbiol. 2022 Sep 28;13:953500. doi: 10.3389/fmicb.2022.953500 (PMC9554406; doi:10.3389/fmicb.2022.953500)
Supplement: SUPPLEMENTARY FIGURE S1 — Diagram explaining how cellular contamination in the metagenomic datasets was assessed and removed. [file Image_1.pdf]

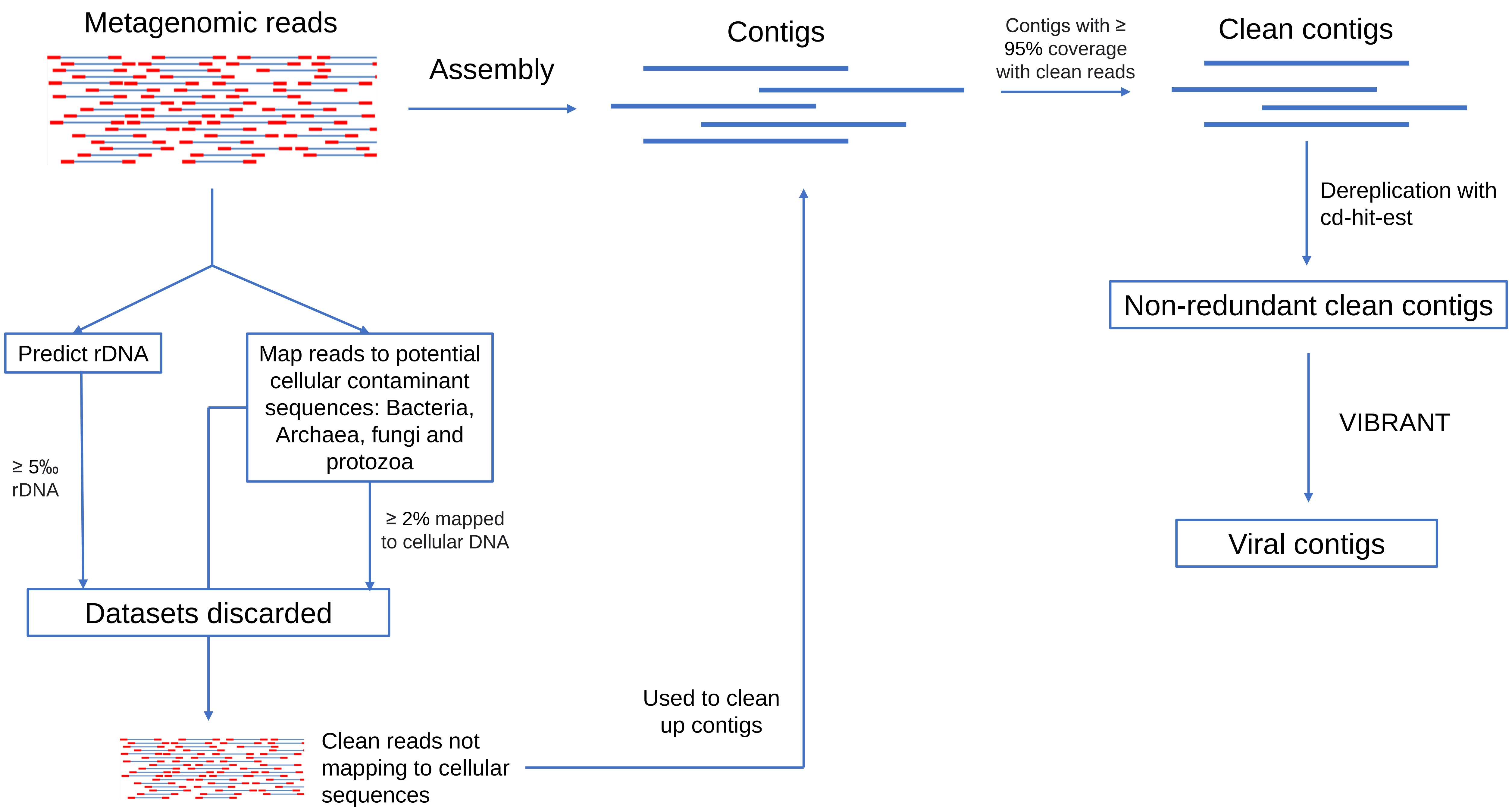

Metagenomic reads
Assembly
Contigs
Contigs with ≥ 95% coverage with clean reads
Clean contigs
Dereplication with cd-hit-est
Non-redundant clean contigs
VIBRANT
Viral contigs
Predict rDNA
Map reads to potential cellular contaminant sequences: Bacteria, Archaea, fungi and protozoa
≥ 5‰ rDNA
≥ 2% mapped to cellular DNA
Datasets discarded
Used to clean up contigs
Clean reads not mapping to cellular sequences
