# Supplementary figures and images for: Insights into the global freshwater virome
Source: Front Microbiol. 2022 Sep 28;13:953500. doi: 10.3389/fmicb.2022.953500 (PMC9554406; doi:10.3389/fmicb.2022.953500)

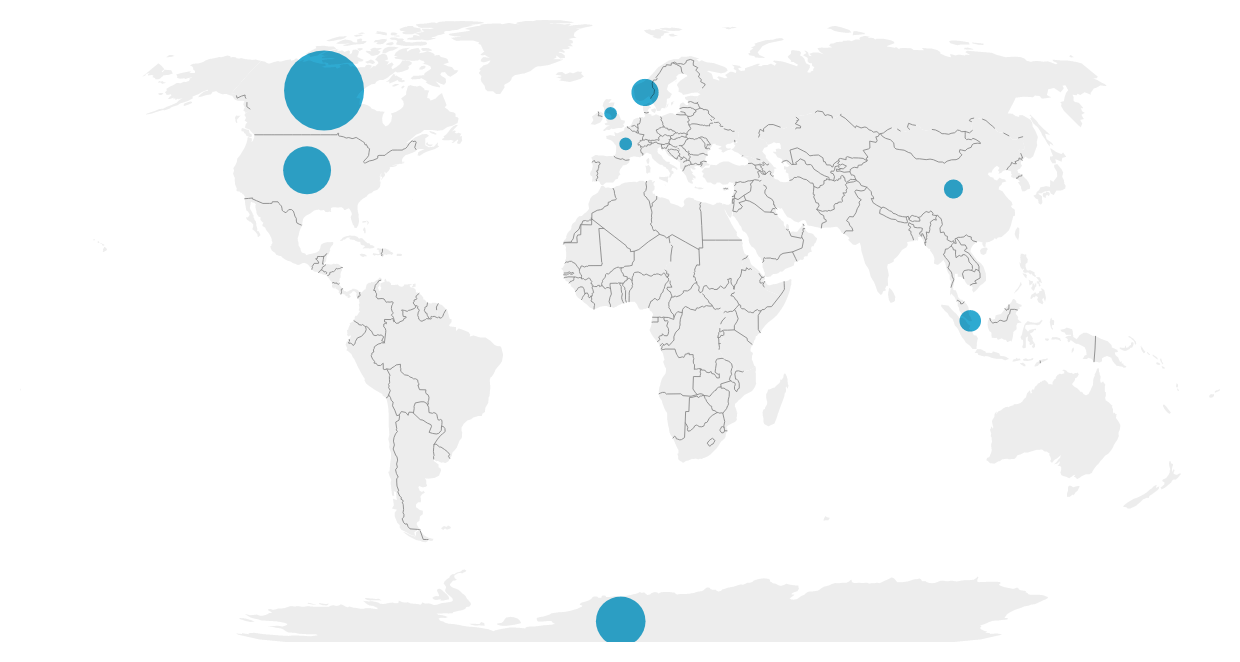

Supplement: SUPPLEMENTARY FIGURE S2 — World map showing the locations of the studied 143 metagenomes. The size of the circles at each location is proportional to the number of metagenomes collected from this location. [file Image_2.png]

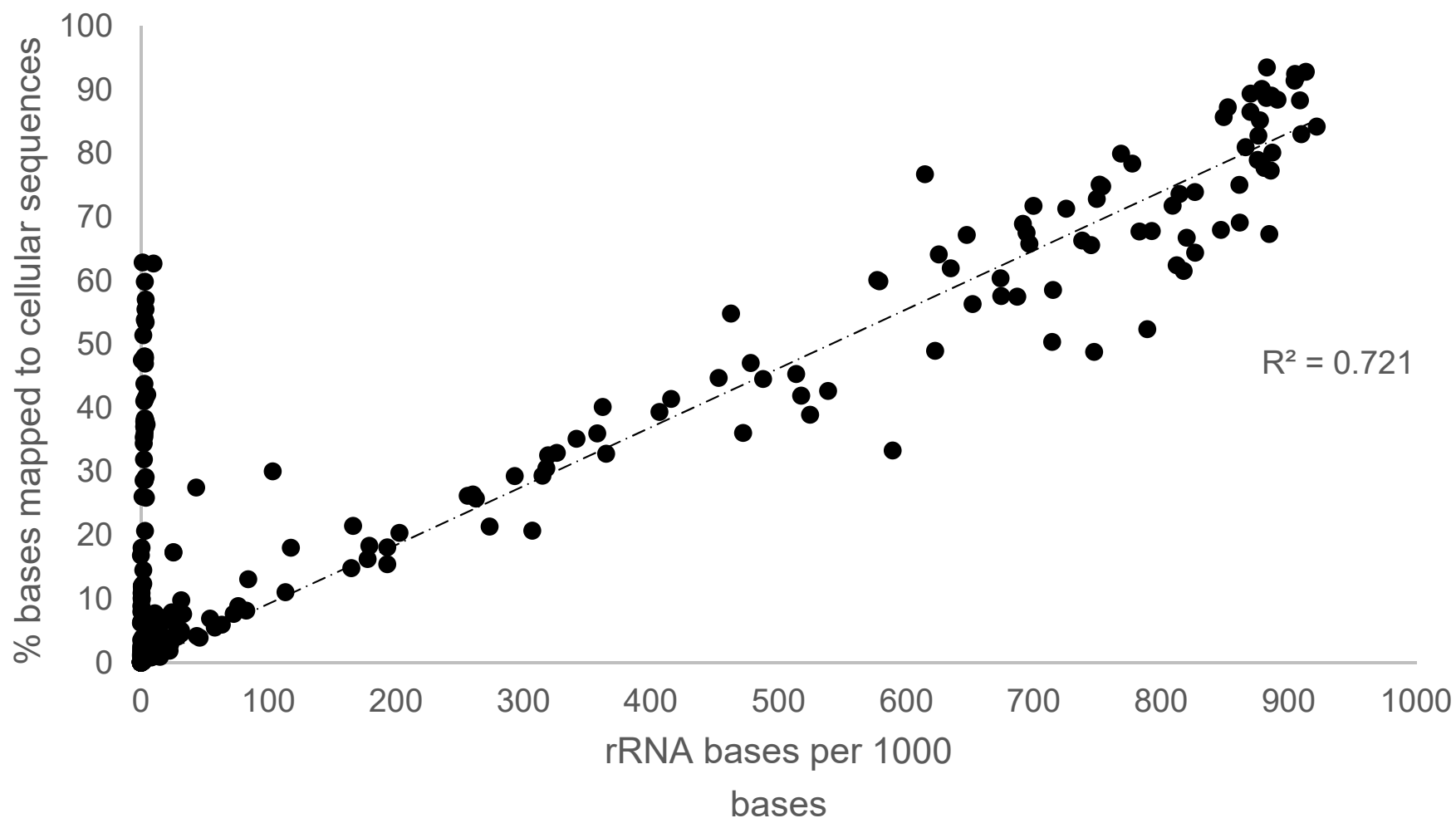

Supplement: SUPPLEMENTARY FIGURE S3 — Percent bases mapped to cellular sequences versus rRNA reads per 1000 reads. [file Image_3.pdf]

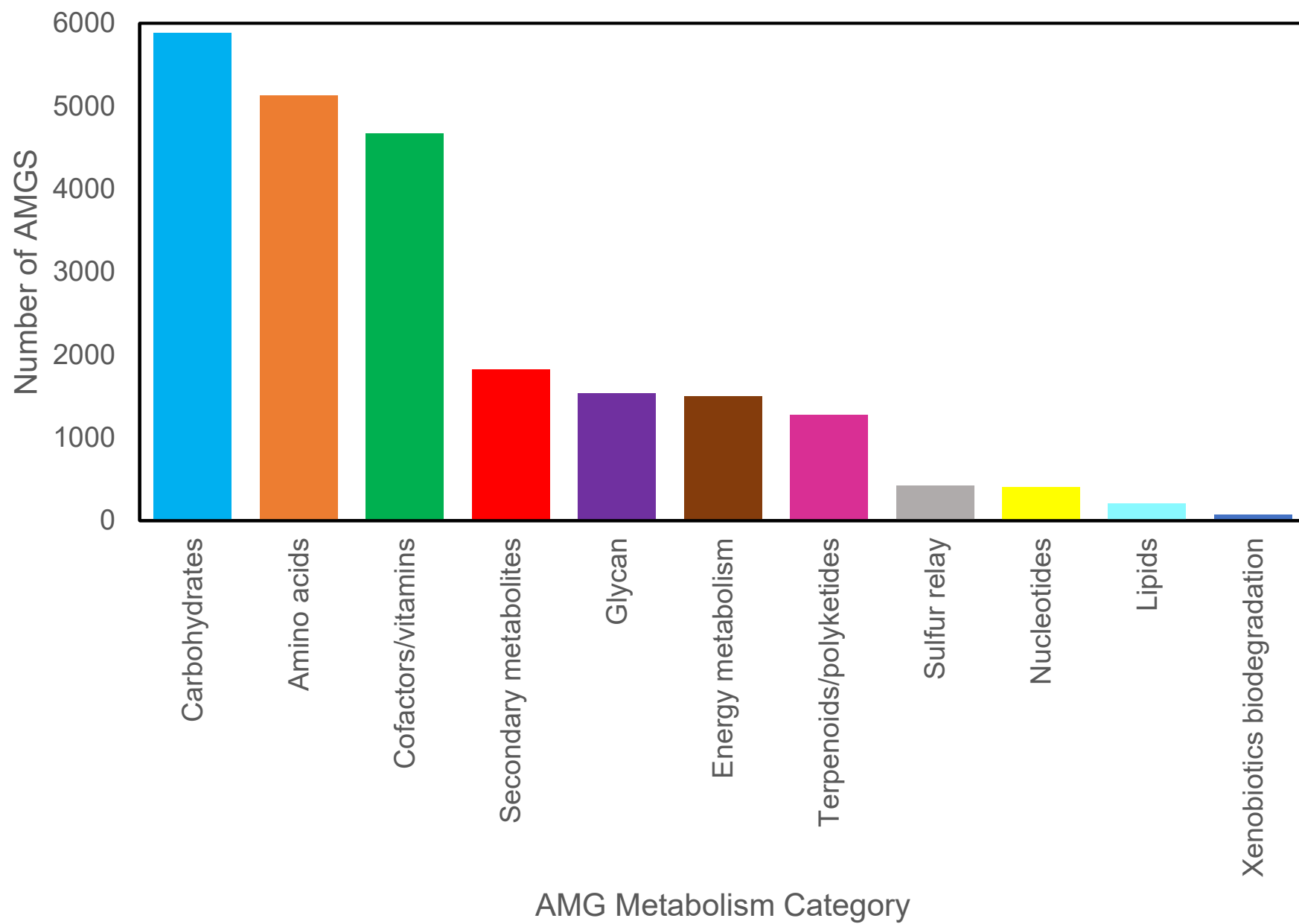

Supplement: SUPPLEMENTARY FIGURE S4 — Numbers of detected AMGs classified according to metabolism categories. [file Image_4.pdf]

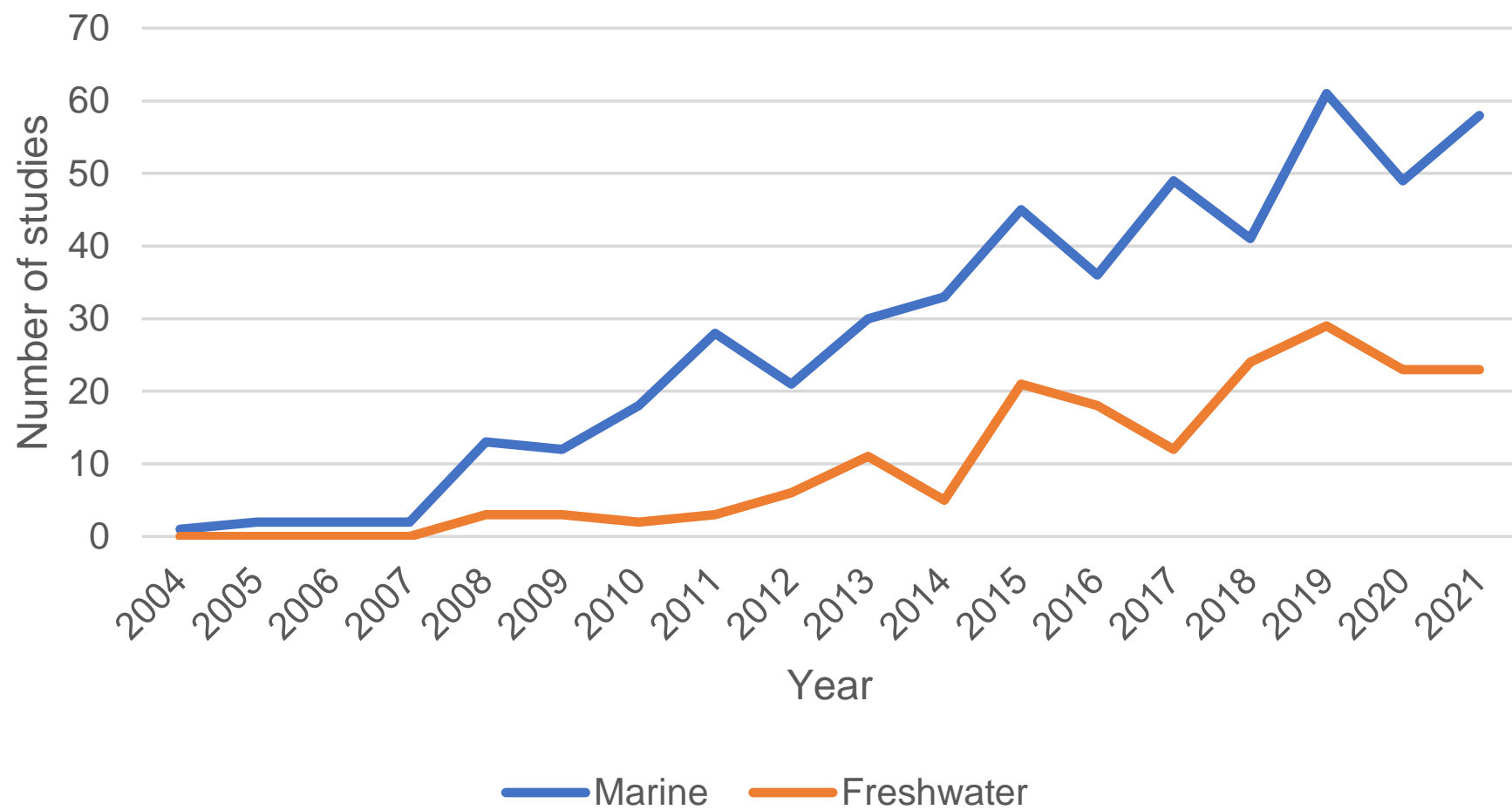

Supplement: SUPPLEMENTARY FIGURE S5 — Annual number of publications for marine viral metagenomes versus freshwater viral metagenomes. [file Image_5.pdf]
